# Spatial and Temporal Lineage Analysis of a Pitx3-Driven Cre-Recombinase Knock-In Mouse Model

**DOI:** 10.1371/journal.pone.0042641

**Published:** 2012-08-01

**Authors:** Marten P. Smidt, Lars von Oerthel, Elisa J. Hoekstra, Raymond D. Schellevis, Marco F. M. Hoekman

**Affiliations:** 1 Molecular Neuroscience, Swammerdam Institute for Life Sciences, University of Amsterdam, Amsterdam, The Netherlands; 2 Department of Neuroscience and Pharmacology, Rudolf Magnus Institute of Neuroscience, University Medical Center Utrecht, Utrecht, The Netherlands; Baylor College of Medicine, United States of America

## Abstract

Development and function of mesodiencephalic dopaminergic (mdDA) neurons has received a lot of scientific interest since these neurons are critically involved in neurological diseases as Parkinson and psychiatric diseases as schizophrenia, depression and attention deficit hyperactivity disorder (ADHD). The understanding of the molecular processes that lead to normal development and function of mdDA neurons has provided insight in the pathology and provided critical information on new treatment paradigms. In order to be able to study specific genetic ablation in mdDA neurons a new tools was developed that drives Cre-recombinase under the control of the Pitx3 locus. The Pitx3 gene is well known for its specific expression in mdDA neurons and is present at the onset of terminal differentiation. Analysis of newly generated Pitx3-Cre knock-in mice shows that Cre expression, measured through the activation of eYfp by removal of a “Stop” signal (LoxP-Stop-LoxP-eYfp reporter mouse), is present at the onset of terminal differentiation and mimics closely the native Pitx3 expression domain. In conclusion, we present here a new Cre-driver mouse model to be used in the restricted ablation of interesting genes in mdDA neurons in order to improve our understanding of the underlying molecular programming.

## Introduction

Mesodiencephalic dopaminergic (mdDA) neurons are involved in voluntary movement control and regulation of emotion related behavior and are affected in many neurological and psychiatric disorders. MdDA neurons form a specific neural group that shares the neurotransmitter identity with several other functionally distinct dopaminergic cell groups in the central nervous system (CNS). The important link between the mdDA neurons in human CNS disorders and behavioral dysfunction has led to the intense study of this neuronal group in pharmacology and more recently also in developmental neurobiology. The former has led to the generation of many pharmacological intervention strategies to alleviate disease symptoms whereas the latter has provided general insight into the origin of the disease and the molecular mechanisms that define mdDA development, physiology and its failure in human pathology. The recent insight into the molecular biology of mdDA neurons [1] has elicited ES-cell replacement strategies [2]–[6] in the treatment of for example Parkinson's disease, where massive mdDA neuronal death occurs. Successful cell replacement strategies start by understanding which molecular programming is essential to generate mdDAneurons, and more specifically how to generate dopaminergic subsets [7]–[9]. Here we report on the generation of a mouse model that is designed to be able to induce Cre-recombinase in these mdDA neurons. To achieve this goal we generated a knock-out/knock-in construct in the Pitx3 locus, a homeobox transcription factor well known for its involvement in mdDA development and for its restricted expression in time (onset of terminal differentiation) and place in these neurons [8]–[12]. The construct was generated by rebuilding a splice acceptor region derived from the mouse En2 gene which was used to generate an exon containing the miCre gene fused to an SV40 poly-A signal sequence. This construct was used to replace part of intron 1 and exon 2–4 of the Pitx3 locus. The resulting Pitx3-Cre mouse was crossed with a LoxP-STOP-LoxP-eYfp reporter line to be able to analyze the Cre expression and efficiency in-vivo. The analysis of these mice clearly showed that the Pitx3-Cre driver is very efficient and induced eYfp in mdDA neurons. The resulting eYfp expression in the adult system covers all mdDA neurons, making it an efficient and reliable tool for the ablation of genes of interest to the development of these neurons.

## Results

### Generation of a new model for mdDA specific expression of Cre-recombinase

**Figure 1:**
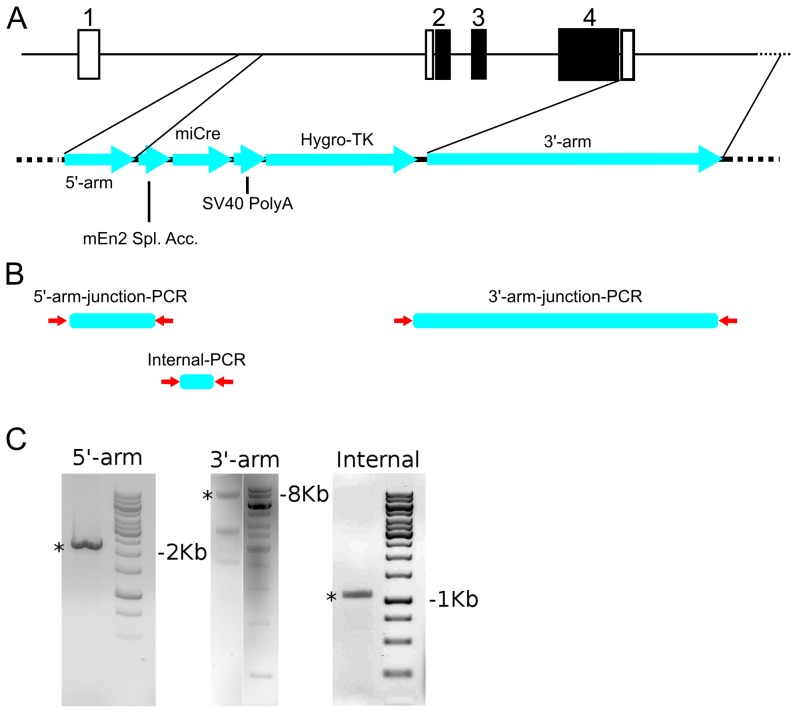
Representation of the construction and validation of the Pitx3-Cre knock-out/knock-in. A) Schematic drawing of the targeting construct design. B) Schematic representation of the construct insertion validation. Primers (red arrows) are chosen as follows: in the 5- flanking region and in the En2 splice acceptor (5-arm-junction-PCR); in the 3′ flanking region and in the Hygo TK sequence (3′-arm-junction-PCR); in the miCRE sequence (Internal-PCR) C) Validation of homologous recombination through PCR analysis using primers outside the construct at the 5′- and 3′ arm and an internal control (miCre). 1–4: Pitx3 exons.; Open squares indicate 5′ and 3′ Utrs; Closed squares indicate coding region.; mEn2 Spl. Acc: mouse En2 Splice acceptor sequence; *: positive Pcr product in genotyping.

There is an enormous interest towards the understanding of the molecular programming of mdDA neurons from a clinical perspective and more fundamental perspective. The former as a tool to generate these neurons for cell-replacement therapy in the treatment of Parkinson's disease (PD) and the latter as a model for molecular programing of neuronal systems in the vertebrate CNS. In order to gain more insight in the role specific transcription factors play in the programming of mdDA neurons, the ablation of such factors at a specific time and place becomes more and more important. To this end we have constructed a Cre knock-in using the mdDA specific gene Pitx3 as a target locus. The construct was generated using a part of the Pitx3 locus that was previously used to generate a Pitx3-Gfp knock-in animal [13], [14]. A Cre containing exon was inserted in the Pitx3 locus (Fig. 1) by cloning an En2 splice acceptor sequence in front of miCRE and an SV40 polyA sequence behind it. This construct was used to induce homologous recombination in ES-cells resulting in an ES-clone containing a proper inserted targeting construct (Fig. 1 B). The resulting ES-clone was injected in blastocysts and chimeric animals were produced. These mice were cross-bred to C57/Bl6 animals and germ-line transmission was established after which these animals were used for further analysis.

### Pitx3 is expressed during development which later mark regions in the lens, cortex, hindbrain and prominently the mesodiencephalon

**Figure 2:**
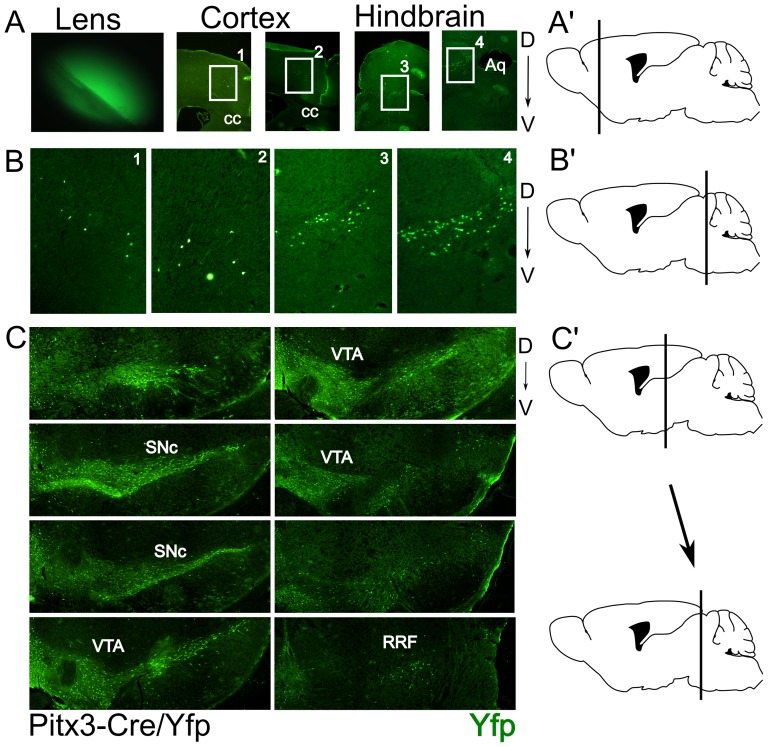
Pitx3-Cre driven expression of eYfp is detected in all mdDA neurons in the adult murine brain. A) Expression of eYfp in the lens and areas of the telencephalon and mesencephalon A′) Schematic drawing of the position of the telencephalic eYfp expression. B) Blow-up figures of the eYfp expression sites as indicated in A. B′) Schematic drawing of the position of the metencephalic eYfp expression. C) Expression of eYfp in the mdDA region. C′) Schematic drawing defining the position between the first section (upper left) and the last section (lower right) of eYfp expression in this region as shown in C. SNc:Substantia Nigra compacta; VTA: Ventral tegmental area; RRF: Retro Rubral Field; D: Dorsal; V: Ventral.

In order to analyze the Cre expression and efficiency we crossed the Pitx3-Cre animals with a LoxP-stop-LoxP-eYfp animal. Direct illumination of the eye of the resulting cross (P0) showed the existence of eYfp in the lens as a result of Cre activity (Fig. 2A), confirming the high and transient expression of Pitx3 in the lens [15], [16]. Brain material was isolated and coronal sections of P30 animals were subjected to immunohistochemistry for eYfp. Subsequent analysis of the material showed that the mdDA region expressed eYfp in high levels (Fig. 2C) suggesting that the initial expression of Pitx3 is restricted to the mdDA neuronal region. In addition to the mesodiencephalic expression, small groups of eYfp positive neurons were found in telencephalic and metencephalic area's (Fig. 2 A/B). Within the telencephalic area the positive neurons appear in an anterior/posterior restricted region of the secondary motor neuron area (Fig. 2A, B1 and 2). In the metencephalic region the area of the sagulum nucleus and the uniform nucleus contain eYfp positive neurons. Until now no expression data has been reported for Pitx3 itself in these latter regions, suggesting that the expression is transient or that the construct has a small influence on the cell-type specific activity of the Pitx3 locus.

### Pitx3 is temporally expressed in non dopaminergic cells in the mesodiencephalon

**Figure 3:**
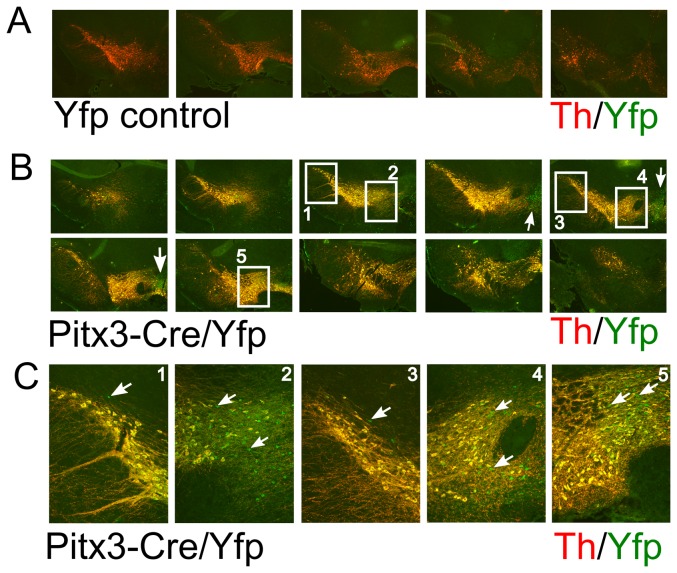
Pitx3-Cre driven expression of eYfp co-localizes with all Th positive neurons. A) Th and eYfp expression in the control LoxP-stop-LoxP-eYfp animal. Note that only Th is expressed in these animals. B) Co-localization of Th and eYfp in the mdDA neuronal region. Note the complete overlap of Th positive neurons with eYfp. C) Enlarged figures of boxes as marked in B. White arrows: indication of the regions and cells where there is eYfp expression with no Th co-localization.

**Figure 4:**
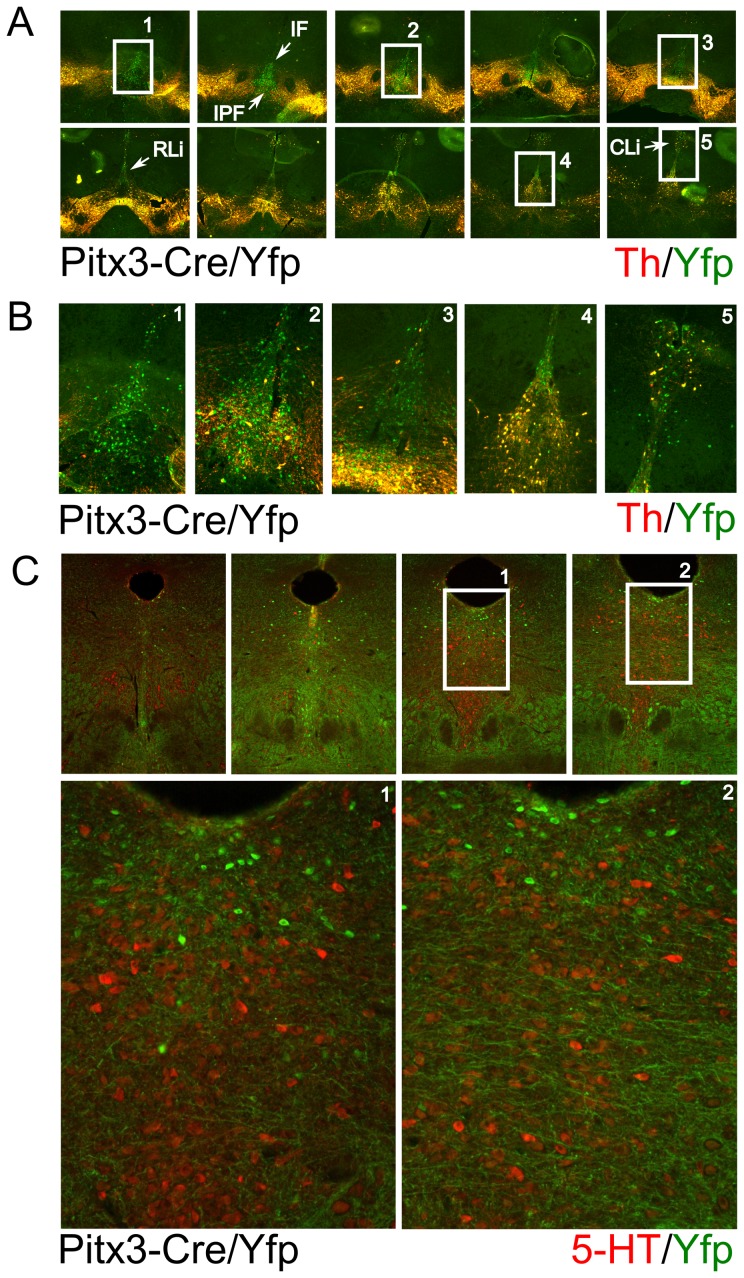
Pitx3-Cre driven expression of eYfp marks transient expression of Pitx3 in a medial non-dopaminergic cell-group. A) In depth analysis through Th and eYfp co-localization of non-dopaminergic regions that express eYfp. B) Enlarged figures of boxes marked in A. C) Co-localistion of Yfp with 5-Ht staining at 4 separate A/P positions from left to right (top 4 panels). Higher magnification of the boxed areas are presented in the lower 2 panels, clearly showing that the Yfp positive neurons do not co-localise with 5-HT neurons. IF: interfascicular nucleus; IPF: interpeduncular fossa; RLi: rostral linear nucleus of the raphe; CLi:caudal linear nucleus of the raphe.

The eYfp signal in the mdDA region suggested that all dopaminergic cells are inducing Cre in time. In order to establish the exact overlap in this region with mdDA neurons we performed double immunohistochemistry for Th and eYfp in Pitx3-Cre/LoxP-stop-LoxP-eYfp and LoxP-stop_loxP-eYfp adult brains (Fig. 3). The LoxP-stop-LoxP-eYfp animals did not show any eYfp signal in the Th expression domain (Fig. 3A), indicating that the eYfp construct does not provide any leakage of the eYfp gene in this region. Moreover, analysis of the whole brain of these control animals did not show any eYfp signal (data not shown). In the Pitx3-Cre/LoxP-stop-LoxP-eYfp brains a clear overlap of Th expression and eYfp was observed and no Th-only positive cells could be detected, indicating that all mdDA cells are exposed to Cre during development as a consequence of Pitx3 gene activation [11]. On the other hand, we did find eYfp positive cells that did not show any Th signal (Fig. 3 B and C, white arrows). These neurons were located at different positions but more prominent in the VTA (Fig. 3 C, white arrows). To fine-map these regions we analyzed these non dopaminergic eYfp positive cells in more detail (Fig. 4). Most of these cells are located in a medial position along the anterior to posterior axis, with the majority of cells in the anterior domain. Mapping of the anatomical locations, mark these eYfp positive cells to the interfascicular nucleus (IF), interpeduncular fossa (IPF), rostral linear nucleus of the raphe (RLi) and caudal linear nucleus of the raphe (CLi) (Fig. 4 A, B). These markings suggest that during development of the mesencephalon, Pitx3 is transiently expressed in neurons that later form these structures. Especially the possibility that Pitx3 is expressed in part of the raphe nucleus indicates that it may play a temporal role in defining the serotonergic phenotype as well as the dopaminergic phenotype. In order to asses whether Pitx3-Cre is (transiently) present in developing 5-HT neurons we performed double labeling of 5-HT and Yfp in adult brain sections of Pitx3-Cre/LoxP-stop-LoxP-eYfp mice. Although the region does contain many Yfp positive neurons, the potential overlap with 5-HT neurons could not be established, suggesting that developing 5-HT neurons in this region are not influenced by Pitx3 expression (Fig. 4C).

### Pitx3-driven Cre expression starts at the onset of mdDA terminal differentiation

**Figure 5:**
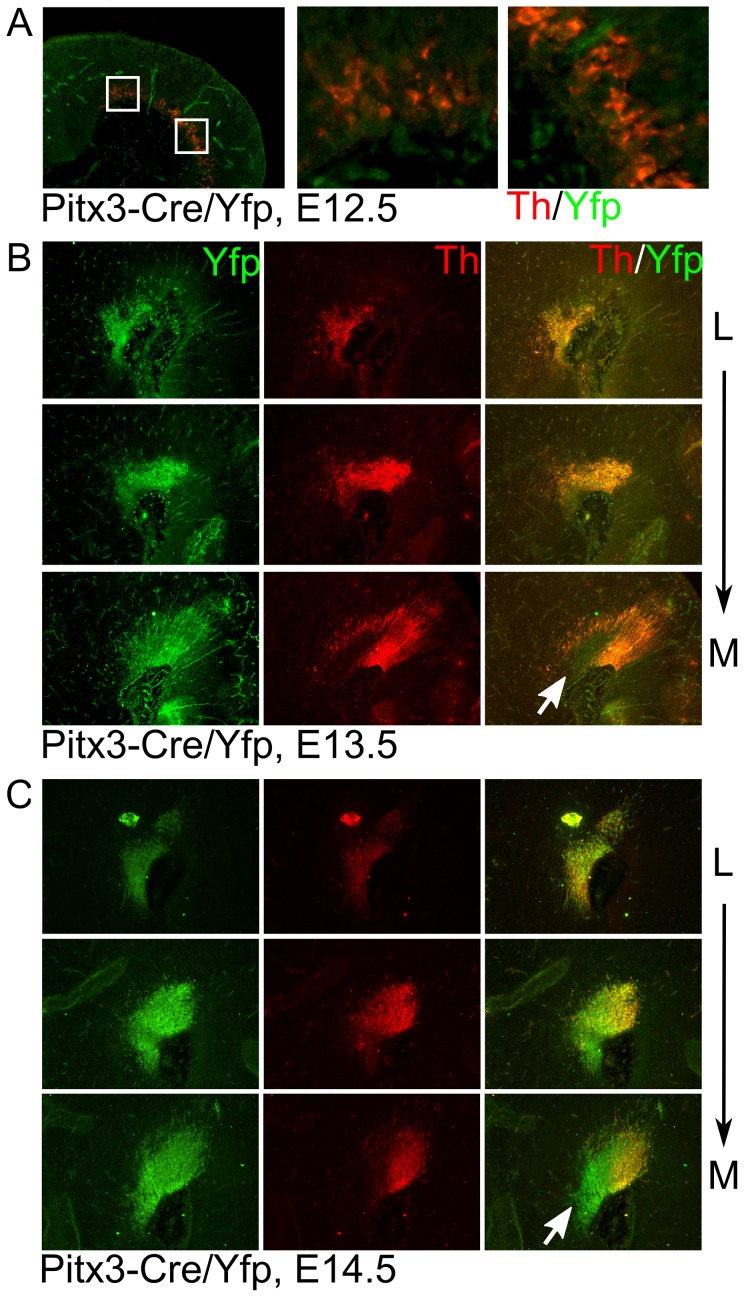
Pitx3-Cre driven eYfp expression starts between E12.5 and E13.5 and marks the ventral tegmentum, overlapping with Th. A) Th and eYfp co-localization in sagittal sections of the ventral mesodiencephalic region. Note that at this stage eYfp is not detected. B) Th and eYfp seperate as well as co-localization in sagittal sections of the ventral mesodiencephalic region. At this stage co-localization can be found but also some areas where Th and eYfp are uncoupled. C) Similar analysis as in B, on E14.5 material, confirming the Yfp and Th distribution. The white arrows indicates a medial/rostral region where Yfp is expressed in cells that do not express Th. L: lateral; M: medial.

Pitx3 locus driven Cre expression should be present in early differentiation dopaminergic neurons along the anterior to posterior axis in time as was described for Pitx3 itself [10], [11]. In order to establish the onset of Cre activity we analyzed Pitx3-Cre/LoxP-stop-LoxP-eYfp embryos at E12.5, E13.5 and E14.5 through Th/eYfp double immunohistochemistry (Fig. 5). At E12.5 a clear area of Th expression could be detected at the ventral mesodiencephalon marking the start of the terminal differentiation of mdDA neurons. At E13.5 a high eYfp signal can be detected that localizes mainly to the most rostral position, co-localizing with Th protein. Interestingly, in the most medial sections at the rostral position the eYfp signal is present but no TH can be detected at this stage. This uncoupling suggests that this area may form the non-DA cell groups that were identified in the adult stage as the (IF) interfascicular nucleus and (IPF) interpeduncular fossa. Next to these cells also dopaminergic cell groups that depend on Pitx3-RA signaling as described [8], might be delayed in the expression of Th in the Pitx3-Cre expression domain. This unique Yfp expression in the medial anterior domain was confirmed at E14.5 (Fig. 5C).

### Pitx3-driven Cre expression is present in embryonic striated muscle

**Figure 6:**
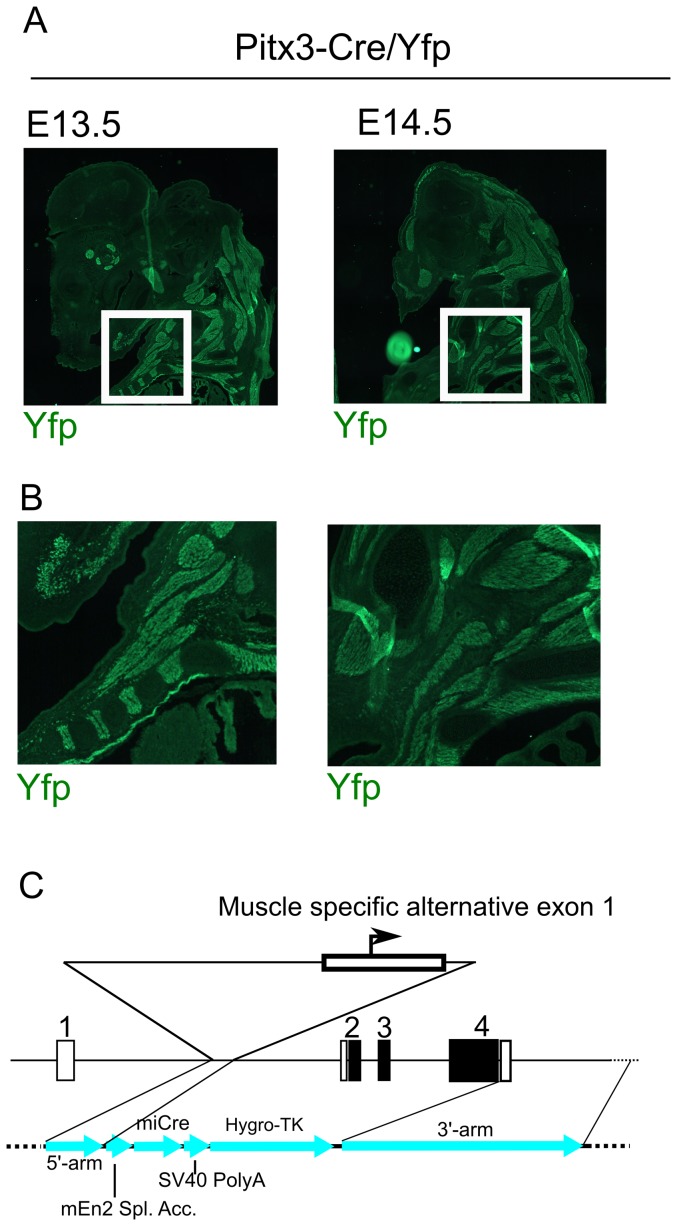
Pitx3-Cre driven eYfp expression outside the brain maps to the muscle. A) Representation of the eYfp muscle expression in the developing embryo at 2 stages of development. B) Enlarged figures of boxes marked in A. C) Schematic representation of the recombinant knock-in construct indicating the position and the 3′-restricted flank of the alternative exon1 position. Figure details as in figure 1.

It has been described that Pitx3 is expressed in embryonic striated muscle and that this expression is mediated through an alternative exon1 in the Pitx3 Intron1 region [10], [17]. Since the Pitx3-Cre construct contains within the 5′-arm the alternative exon 1 and is restricted directly after this exon we have analyzed the expression of eYfp inPitx3-Cre/LoxP-stop-LoxP-eYfp animals to confirm the transgenic approach that was the basis of the identification of this controller region [17]. Through immunohistochemistry for eYfp in sagittal sections of E13.5 and E14.5Pitx3-Cre/LoxP-stop-LoxP-eYfp embryos we were able to show that most striated muscle cells contain eYfp and therefore express Cre at least at similar onset as found within the brain (Fig. 6). The ablation of any intron sequences beyond the alternative exon1 (Fig. 6 C) proves that the proposed sequence is able to drive striated muscle expression [17] in the endogenous in-vivo situation.

## Discussion

### Pitx3-Cre: a novel tool for induction of Cre in all mdDA neurons

Due to the enormous interest towards the understanding of the molecular programming of mdDA neurons we here report on the generation of a new tool to induce the ablation of critical factors in mdDA neurons at the start of terminal differentiation. Already available tools as the Th-Cre (Bac-transgenic [18]) and the Dat-Cre (knock-out/knock-in [19], [20]) are different in position and timing aspects of expression. The Th-Cre induces Cre in all catecholaminergic cell-groups in the CNS and the timing and position in mdDA neurons is different due to the dependency of Th expression to Pitx3-RA signaling, especially in the SNc region. This means that Pitx3-Cre will provide a more restricted and possibly more complete Cre induction pattern in mdDA neurons as compared to Th-Cre. The Dat-Cre model provides Cre expression at a later stage since the onset of Dat itself is around E13.5 (mouse) and is more prominent in rostral mdDA neurons. This means that the Cre induction is very low or even absent in parts of the VTA. The exact timing however and possible distribution of the Dat-Cre has not been analyzed in detail in the original paper [19]. Another complicating factor is the fact that this Dat-Cre animal has a deletion of the Dat gene, generating a phenotype in mdDA neurons by itself. This issue was solved through the generation of a Dat-Cre knock-in, in which the Cre gene was positioned in the 3′-Utr of the Dat locus. In this way the gene dosage of Dat was not affected [20]. The analysis through induction of a LacZ construct has, in our view, not been performed in enough detail to be able to judge the amount of overlap with mdDA neurons in the VTA. This leaves the issue under discussion whether this model will provide enough Cre expression in the VTA to be able to remove all floxed target genes in these neurons. The generation of a Pitx3-Cre driver model will provide a valuable genetic tool to be able to induce specific mutations of “floxed” genes in all mdDA neurons as we have shown here. Due to the fact that a knock-in strategy was used where the Pitx3 gene was removed, the transgene is stable and can even be used to make double inducible knock-out lines where interaction with Pitx3 itself may be analyzed. The ablation of one allele of Pitx3 is not a problem in terms of coding defects. In earlier studies we have shown that there is only a minor effect on Ahd2 expression due to the fact that this gene is directly regulated by Pitx3 in mdDA neurons [8], [9].

### Pitx3-driven Cre expression maps to all mdDA neurons

In order to ensure that Cre expression is present in all mdDA neurons we have mapped Cre activity through an eYfp reporter. The data clearly shows that the overlap with Th expression in the adult mdDA system is complete. This makes the Pitx3-Cre driver an ideal tool for the mutation of “floxed” genes in mdDA neurons. Interestingly, the co-localization with Th is not complete during development as was shown earlier for the Pitx3-Gfp mouse model [13], [14]. The temporal dissociation of Th and Cre is due to molecular coding differences that generate specific mdDA populations as exemplified through specific Th activation mechanisms [8], [9], [12]. In addition to the mdDA neurons we did locate eYfp expression as a consequence of Pitx3-Cre activation in small areas outside mdDA neurons. Some cells in the prefrontal cortex and hindbrain were mapped. These areas were not identified until now for their (temporal) expression of Pitx3. It remains to be determined whether this is a consequence of the Pitx3 locus alteration (inclusion of *Hygro-Tk* in the locus) or that Pitx3 is natively activated in these neurons at some point during development. The most prominent extra mdDA neuronal expression of eYfp (Cre) was noted close to these neurons within the VTA and SNc region and interestingly in areas mapping to the raphe nucleus. However, no clear co-localization of eYfp and 5-HT staining could be determined. It is possible that Pitx3 is involved in the specification of interneurons in these areas in analogy to the induction of Gabaergic inter-neurons by the C. elegans homologue Unc30 [21], [22].

### The alternative Pitx3 exon1 drives Cre expression towards muscle in-vivo

The peripheral expression of Pitx3 was described to be present in a temporal fashion in the eye lens and the muscle [10], [17]. We have shown that the lens expression is conserved in the Pitx3-Cre model as well as the muscle expression. The latter Cre induction is interesting since the original paper described a transgenic approach and mapped the sequence essential for the induction of Pitx3 in striated muscle tissue [17]. Analysis of our knock-in animal model enabled us to support their data, suggesting that the sequence earlier identified is essential and sufficient in a normal in-vivo context to drive striated muscle expression. Taken together, the novel Pitx3-Cre mouse model as presented here can be used to induce mutation of “floxed” genes in all mdDA neurons and thus provides and essential new tool in the ongoing study into the molecular programming of mdDA neurons in health and disease.

## Materials and Methods

### Ethics statement

All animal studies are performed in accordance with local animal welfare regulations, as this project has been approved by the animal experimental committee (Dier ethische commissie Universitair medisch centrum Utrecht; DEC-UMC-U) and international guidelines.

### Cloning

The En2 splice acceptor sequence (ncbi gi 403021) was generated through PCR of the mouse genome using the following primers:5′ GAGGGATCCTCGAGCCAGCAACCAGTAACCTCTG 3′; 5′ GAGAAGCTTGCTCCTGTGCCAGACTCTGG3′. The miCre cDNA was sub-cloned from a plasmid (kind gift of Ruud Toonen, The Netherlands) by PCR using the following primers:5′ ATCAAGCTTGTCCACCATGG 3′; 5′ CTCGAATTCAGTCCCCATCTTCGA GCA G 3′. The SV40 Poly A sequence was cloned through PCR from the pSG5 plasmid using the following primers: 5′ GGCGAATTCGGATCCAGATCTT3′ ; 5′ ATAGAATTCTCTAGAGTCGACCAGACATG 3′. Fragment were subsequently cloned into Pgem7 to generate the En2-splice acceptor-ICRE-SV40 poly-A part. This fragment was cloned into the Pitx3 locus containing the 5′-arm and the3′-arm of Pitx3 (kind gift of Meng Li, UK [13]). All sub-cloning steps and the final cloning of the targeting construct were sequenced to ensure the validity of the material.

### ES targeting and blastocyst injections

ES-targeting and blastocyst injections were performed as described (transgenic facility of the University of Utrecht [23]) and in accordance with local animal welfare regulations.

### Genotyping

Genotypes of Pitx3-Cre ES cells or animals were determined by PCR (ELT-kit (Roche) analysis as follows:5′-arm PCR primers:

5′ CCACCATAATGCCAGTGAAG 3′;

5′GAGAAGCTTGCTCCTGTGCCAGACTCTGG 3′ expected length ∼2.3 kb

3′-arm PCR primers:

5′ TCAGGGTCATCCTTGGCTAC 3′;

5′ TCGGGGACACGTTATTTACC 3′; expected length ∼8 kb.

Within the construct (iCre):

5′ ATCAAGCTTGTCCACCATG G 3′;

5′CTCGAATTCAGTCCCCATCTTCGAGCAG 3′, expected length ∼1,2 kb

### Animals

Initial chimeric animals were cross-bred to C57/Bl6 animals, selected for germ-line transmission and kept as heterozygotes(F1). Heterozygous (F1) Pitx3-Cre animals were crossed with a transgenic LoxP-Stop-LoxP-eYfp reporter line (B6.129X1-Gt(ROSA)26Sortm1(EYFP)Cos/J, Jaxmice no. 006148) to generate Pitx3-Cre/loxP-Stop-LoxP-eYfp animals for analysis, all in accordance with local animal welfare regulations.

### Immunohistochemistry

Embryos or adult brains were isolated, fixed in 4% PFA overnight, washed in 1*PBS, incubated in 30% sucrose/PBS for 24 hours and frozen on dry ice. For immunostaining, sections were washed three times in 1*TBS and blocked for 30 minutes in TBS with 4% hiFCS. Primary antibodies in THZT were applied overnight at 4 degrees C. and slices were washed three times in 1*TBS. Secondary antibodies were applied in 1*TBS for 1 hour at room temperature. Slices were then washed three times in 1*PBS and mounted using FluorSave Reagent (Merck). Primary antibodies used were chicken anti-GFP (1∶500, Abcam), rabbit anti 5-HT (1∶5000; kind gift of T. Vitalis) and rabbit anti-TH (1∶1000, Pellfreeze). Secondary antibodies (1∶400) used were goat anti-chicken (Alexa 488, Invitrogen), goat anti-rabbit (Alexa 594, Invitrogen).
